# Is L-lactate a novel signaling molecule in the brain?

**DOI:** 10.1038/jcbfm.2015.77

**Published:** 2015-04-29

**Authors:** Valentina Mosienko, Anja G Teschemacher, Sergey Kasparov

**Affiliations:** 1 School of Physiology and Pharmacology, University of Bristol, Bristol, UK

**Keywords:** astrocytes, ATP, brain slice, lactate, optogenetics

## Abstract

In the brain, L-lactate is produced by both neurons and astrocytes. There is no doubt that neurons use L-lactate as a supplementary fuel although the importance of this energy source is disputed. Irrespective of its caloric value, L-lactate might also have a signaling role in the brain. Here, we review several current hypotheses of L-lactate mediated signaling. Some proposed mechanisms require L-lactate entry into the neurons leading to a shift in ATP/ADP ratio or redox state. Others postulate interaction with either known receptor HCA1 (GPR81) or a novel, yet unidentified receptor. We argue that the sensitivity of any such mechanism has to match the concentration range of extracellular L-lactate, which is less than ~1.5 mmol/L under physiologic conditions. From that point of view, some of the proposed mechanisms require supraphysiologic levels of L-lactate and could be engaged during ischemia or seizures when L-lactate concentration rises dramatically. Currently, we do not know whether L-lactate production in the brain occurs in microdomains, which might create higher than average local concentrations. Nevertheless, it is clear that in the brain, as in the peripheral tissues, L-lactate is not only used as a source of energy but also acts as a signaling molecule.

## Introduction

L-lactate is one of the most common metabolites in the mammalian body and is produced in all cells including neurons and glia of the brain. Glycolysis leads to formation of pyruvate, part of which is converted into L-lactate by lactate dehydrogenase (LDH). Very good and extensive recent reviews are available on the topic of L-lactate metabolism in the central nervous system (CNS)^1, 2^ where L-lactate traditionally has been seen as an alternative to glucose energy source. However, several studies have suggested that, in addition to this role, L-lactate might also act as a true signaling molecule and alters the activity of neurons *via* a variety of mechanisms. In this review, we summarize some of the evidence that implicates L-lactate in intercellular signaling in the brain.

## Operational range and sources of L-lactate in the brain

While L-lactate can reach the brain from the periphery, under normal conditions, L-lactate influx from the bloodstream into the CNS is fairly limited due to the blood–brain barrier and a small concentration gradient. Boumezbeur *et al*^3^ combined [3-^13^C] labeled L-lactate with ^13^C magnetic resonance spectroscopy to study L-lactate kinetics and metabolism *in vivo* in young healthy volunteers. After an overnight fasting subjects were administered L-lactate infusions to maintain plasma levels at either ~1.5  or ~2.5 mmol/L (approximately double of the estimated resting L-lactate level). At 1.5 mmol/L plasma L-lactate concentration, there was almost no net L-lactate consumption in the brain but this was increased when plasma L-lactate was raised. Using mathematical modeling the authors suggested that L-lactate might contribute ~10% to the brain metabolism at normal physiologic peripheral levels but its contribution could increase to ~60% with supra-physiologic plasma L-lactate concentrations.

**Figure 1:**
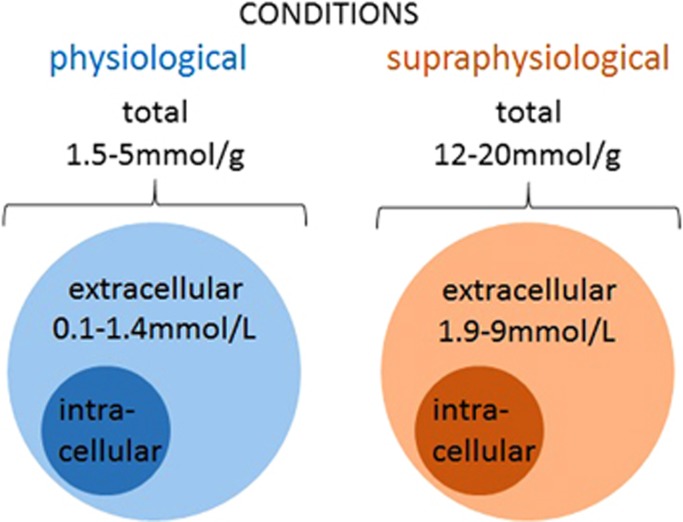
L-lactate levels in the brain under normal and supraphysiologic conditions. Central L-lactate content depends on various conditions, and reaches 1.5 to 5 *μ*mol/g under physiologic conditions, and increases up to 12 to 20 *μ*mol/g with aging or under supraphysiologic conditions such as hypoxia or hyperglycemia. Extracellular L-lactate level in the brain as measured by microdialysis is reported to vary from ~0.1 to 0.35 mmol/L in freely moving rats, up to 1 to 1.6 mmol/L in rat hippocampus and striatum, and increases up to 9 mmol/L after convulsive shock or ischemia. In humans, extracellular L-lactate levels in the cortex were reported to be ~1 mmol/L.

Several studies have estimated central L-lactate concentrations and shown that total L-lactate content in the CNS depends on plasma glucose levels as well as on physiologic conditions (e.g., normoxia vs hypoxia or increased excitability) and concentrations fluctuate independently of peripheral levels (Figure 1). In mice, total L-lactate content in the cerebral cortex was reported to increase from ~5  to 12 *μ*mol/g with aging.^4^ These values however need to be taken with caution because they were measured in brain tissue from animals, which had to be killed and then processed for tissue extraction. It is more than likely that hypoxia after the cessation of respiration could have led to a significant rise in lactate levels. In rats, under normal conditions total L-lactate content in the supratentorial part of the brain was close to 1.5 *μ*mol/g of tissue weight^5^ but could reach up to 20 *μ*mol/g after ischemia or during hyperglycemia.^6, 7^ When measured by microdialysis in the posterior hippocampus, under normal conditions extracellular L-lactate was ~1.4 mmol/L, but increased to ~3.5 mmol/L during hypoxia.^8^ Another study reported a similar concentration of extracellular L-lactate in striatum (1 mmol/L), which increased to ~1.9 mmol/L after 2 minutes of electroconvulsive shock.^9^ In the dorsolateral striatum, extracellular L-lactate concentration was ~0.6 mmol/L at rest but increased to up to 9 mmol/L after 5 minutes of ischemia.^10^ Two other studies reported lower basal levels of extracellular L-lactate in striatum of freely moving rats (~0.1 to 0.25 mmol/L according to Fray *et al*^11^ and ~0.35 mmol/L according to Demestre *et al*^12^). Finally, a very recent publication used a novel approach to estimate the intracellular level of L-lactate *in vivo*.^13^ In that study the authors employed a novel genetically encoded ratiometric sensor for L-lactate, called Laconic.^14^ Laconic was expressed in the cortical astrocytes of mice or *in vitro* in cultured astrocytes and various manipulations were used to estimate concentration of L-lactate inside astrocytes at rest and after procedures aimed to increase or decrease L-lactate production or release. This study reported the basal level of L-lactate within astrocytes to be around 1.4 mmol/L, which is probably an underestimate. Interestingly, electrical stimulation of the tissue triggered an instant decrease in L-lactate concentration within astrocytes, which most likely was due to the quick release of L-lactate. After a few seconds, it was followed by a rise in L-lactate (probably a reflection of rapid upregulation of glycolysis). These experiments clearly show that in the intact brain there is a glial reservoir of L-lactate, which can be mobilized within milliseconds upon activation.^13^

In human patients undergoing surgery after traumatic brain injury, microdialysis probes placed in a healthy part of the cortex revealed extracellular L-lactate concentrations of 0.73 mmol/L,^15^ and of 1.4 mmol/L.^16^ The difference in perfusion rate, 1 *μ*L/min^15^ and 2 *μ*L/min,^16^ might have contributed to the observed differences in L-lactate levels. Interestingly, the extracellular L-lactate level was not different during anesthesia and awake states, and increased by 50% during transient ischemic episodes through which some of the patients went after the surgery.^16^ However, values of the extracellular L-lactate concentration from these studies need to be taken with some caution because they could be affected by concomitant treatment which these patients received. Regional differences in L-lactate concentrations in human and animal studies are also likely to exist.

Using *in vivo* ^1^H nuclear magnetic resonance, a noninvasive method to observe metabolite changes, a twofold increase in brain L-lactate level was detected during visual stimulation in humans although absolute concentrations of extracellular L-lactate could not be reliably derived from these measurements.^6^

To summarize, extracellular concentration of L-lactate in the brain may be estimated to fluctuate between a few hundreds of *μ*mol/L to 1 to 2 mmol/L and possibly rise to further to several mmol/L under pathologic conditions such as seizures and ischemia (Figure 1).

L-lactate is a metabolite which is not stored in exocytotic vesicles, and exit of L-lactate from the cells which produce it, is passive, which implies that L-lactate appears in the extracellular space under the pressure of the intra-extracellular concentration gradient. Therefore, when considering L-lactate as neuro- or glio-transmitter, we need to assume that signaling should occur within the range of concentrations detectable in the extracellular space of the brain rather than in highly localized transients, as is the case for synaptic transmitter release, for example of glutamate or GABA. Moreover, synthesis rather than release may then be expected to constitute the rate-limiting step for signaling.

## The L-lactate shuttle hypothesis and major sources of extracellular L-lactate in activated brain

L-lactate is well known for its ability to move between cellular compartments, different cells and indeed different organs within the body (reviewed in^17, 18^). This concept extended to the CNS is the basis for the astrocyte-to-neuron L-lactate shuttle hypothesis:^19^ Approximately two decades ago, it was proposed that astrocytes, due to their ability to handle glucose fluxes over the blood–brain barrier, can release L-lactate which they supply as an essential energy source to the adjacent neurons *in situations* of rising energy demand.^20, 21, 22^ Moreover, the sole energy reserve of the brain, glycogen, is stored in astrocytes and can be used when the metabolic demand is high.^23, 24^

The LDH isoenzyme 5, LDH5, which favors L-lactate production, is enriched in astrocytes. Neurons express mostly LDH1, which preferentially supports the L-lactate utilization pathway and, in theory, this distribution should aid neurons in using astrocyte-derived L-lactate as an energy substrate.^25^ Interestingly, in theory astrocytes take up L-lactate from extracellular space 4.3-fold faster and with 2.3-fold higher capacity compared with neurons.^26^ However, since the kinetic properties of these two isoenzymes were mostly measured at 20°C to 25°C, and since the equilibrium constant (*K*_eq_) of these enzymes is the same as they catalyze the same chemical reaction, the preference of LDH isoenzymes over lactate or pyruvate under steady-state conditions is still debated.^27, 28^ One possible scenario in which differences in LDH isoenzymes could play a role is brain activation, when an increase in metabolism leads to increased pyruvate production that potentially shifts an equilibrium of LDH reaction and facilitates pyruvate utilization *via* LDH5.^27, 28^

Whether release of L-lactate from astrocytes may be compartmentalized remains an unresolved issue. Experiments with ammonia-evoked L-lactate release^29^ suggested that glycolysis, and therefore L-lactate production and release into the blood or toward other targets, might be specifically upregulated in perivascular astrocytic endfeet.

Transport of L-lactate into/out of neuronal or glial cells is assisted mainly by monocarboxylate transporters (MCTs). Monocarboxylate transporters cotransport monocarboxylates and protons by a symport mechanism with 1:1 stoichiometry. Transport can be enhanced by acidifying the side from where L-lactate is supplied.^30^ Multiple types of MCT have been identified^31^ but only MCT1–MCT4 have the ability to transport L-lactate.^32, 33, 34, 35^ MCT4 has a relatively low affinity but high transport rate and is thought to be the major MCT of astrocytes.^36, 37^ In contrast, the high-affinity transporter MCT2 is most likely to be neuron specific.^38, 39^ Although some earlier immunohistochemical studies reported that rat brain astrocytes express MCT2,^40, 41^ later work showed that the antibodies used in those experiments were not sufficiently specific and only confirmed localization of MCT2 in neurons.^36^ In addition, the intermediate affinity MCT1 was found in cultured astrocytes,^38^ but later studies claimed that it is only present in blood vessels.^17^ In fact, not only astrocytes but also neurons produce and release L-lactate, the main difference being that neuronal transporters would probably be saturated at relatively low L-lactate levels, making neurons poor exporters of L-lactate.^1^ In theory, this should also make them poor importers of lactate. This differential distribution of MCT4 (predominantly on astrocytes) vs MTC2 (predominantly on neurons) should, again, facilitate the flux of L-lactate from astrocytes to neurons, a situation which may be compared with skeletal muscle where MTC4 is abundant on glycolytic muscle fibers that export L-lactate while MCT1 is expressed by oxidative muscle cells that import L-lactate.^17^ A recent review^42^ summarizes the data on colocalization and functions of the MCT1, MCT2, and MCT4 and discusses their possible roles in lactate transport at rest and during exercise.

Several studies noted a decrease in intracellular pH after increasing extracellular L-lactate concentration in astrocyte cultures^43^ or oocytes expressing MCT1.^44^ Interestingly, such a change in pH was not abolished by MCT blockers, suggesting an alternative pathway for intracellular L-lactate transport. It is important to realize that entry of L-lactate *via* MCT inevitably leads to acidification even when pH-neutral L-lactate solutions are used because of the cotransport of protons into the cell by all MCTs. This should be taken into account when interpreting various experiments.

In support of the L-lactate shuttle hypothesis under conditions of increased brain activity, it was shown that glutamate released at excitatory synapses can enter astrocytes in a Na^+^-dependent manner, promoting glucose consumption, glycolysis, and production of L-lactate that can then be provided to the neurons.^45^ Furthermore, one study suggested that high rates of glycolysis such as required to support sustained levels of activity could be toxic to neurons. The same paper reported also that neurons limit glycolysis by actively degrading 6-phosphofructo-2-kinase (Pfkfb3), a critical enzyme in the glycolytic glucose consumption pathway, and instead actively use the pentose phosphate pathway. This would limit the ability of neurons to produce large quantities of L-lactate.^46^ By extension, L-lactate that rapidly appears in the brain during activation should be mainly of astrocytic origin.

Several studies have showed that interfering with L-lactate production or transport at critical time points in learning paradigms has a negative impact on memory formation, a clear indication of the biologic relevance of an increased L-lactate production on demand.^47, 48, 49, 50^ Curiously, build-up of the D-isomer of L-lactate in the blood as a result of intestinal pathology has a negative effect on memory.^51^

However, the idea that these effects may be easily explained by the loss of the caloric value of imported L-lactate for neurons has been challenged by a number of publications, which argue that in the presence of glucose (as may be expected under physiologic conditions) neurons do not rely on alternative fuels such as L-lactate.^52^ Along the same line, it has been suggested that increased glycogenolysis in astrocytes during periods of increased local brain activity rather helps to sustain the astrocytes themselves and to prevent their rising demand for glucose so that more glucose is left for the metabolically challenged neurons.^26, 53^

To summarize, while the differential distribution of transporters, the presence of glycogen only in astrocytes, a possible cap on upregulation of glycolysis in neurons and other data are generally consistent with the idea that astrocyte-derived L-lactate could be available to neurons as a source of energy, there is no consensus on how biologically important this additional resource actually is. Could L-lactate release and build-up in extracellular space have other but metabolic effects? Could L-lactate be used for intercellular signaling?

## L-lactate as a signaling molecule

### The L-lactate Receptor GPR81 (HCA1)

Sequencing of human and other genomes has made it possible to scan protein-coding parts using algorithms designed to discover putative G protein-coupled receptors (GPCRs). These remarkable proteins occupy large portions of mammalian genomes and have a number of highly conserved structural determinants including their seven transmembrane domain structure with extracellular amino- and intracellular carboxy-terminals. GPR81 (or hydroxycarboxylic receptor 1, HCA1) was one of a number of such proteins initially classified as orphan receptors.^54^ In 2008 and 2009, two groups reported that L-lactate is a natural ligand and agonist of GPR81.^55, 56^ Monocarboxylates alpha-hydroxybutyrate, glycolate, alpha-hydroxyisobutyrate, and gamma-hydroxybutyrate were also identified as GPR81 agonists. In contrast, L-lactate was unable to activate the highly homologous GPCRs, GPR109a (HCA2) and GPR109b (HCA3).^55, 57^

**Figure 2:**
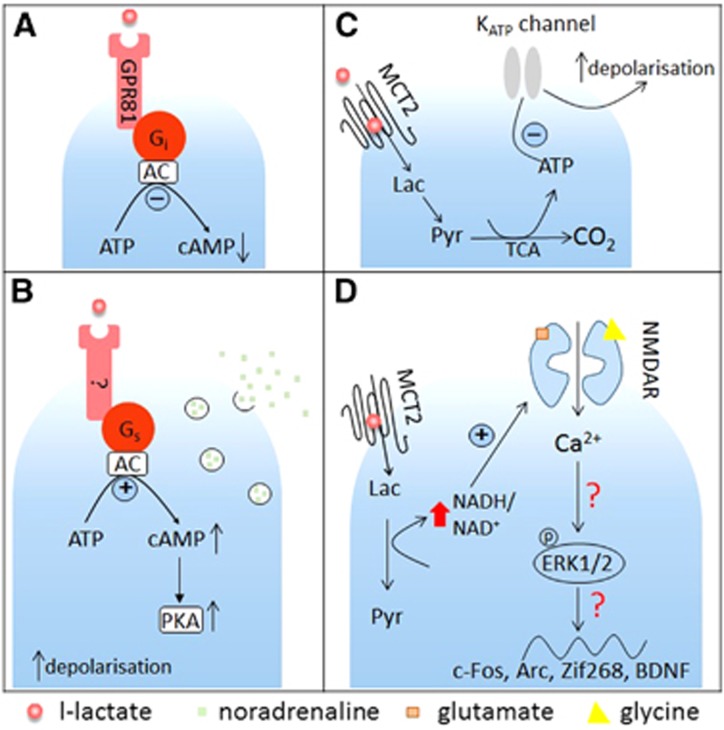
L-lactate-mediated signaling in the central nervous system. (**A**) GPR81-mediated signaling. GPR81 (HCA1) was reported to be expressed in the brain, mostly in neurons. It couples to G_i_ proteins, leading to adenylate cyclase (AC) inhibition and reduced cAMP production. L-lactate activates GPR81 at concentrations above 2.5 mmol/l (studies often use 5 to 10 mmol/L). (**B**) G_s_ protein-coupled receptor mediated signaling. Astrocyte-derived L-lactate excites noradrenergic cells in the locus coeruleus in a concentration-dependent manner. This effect is mediated by a putative G_s_-coupled receptor, and accompanied with accumulation of cAMP and activation of protein kinase A (PKA). Estimated EC_50_ for this effect of L-lactate was ~600 *μ*mol/L. (**C**) K_ATP_ channel-mediated signaling in orexin neurons. Astrocyte-derived L-lactate enters neurons through monocarboxylate transporter 2 (MCT2), is metabolized to pyruvate and used for generation of ATP in tricarboxylic acid (TCA) cycle. This causes a shift in intracellular ATP/ADP ratio, this closes K_ATP_ channels leading to membrane depolarization and neuronal excitation. (**D**) NMDA receptor-modulated signaling. L-lactate transported by MCT2 into hippocampal neurons is converted into pyruvate, causing an increase in intracellular NADH/NAD^+^ ratio that results in potentiation of NMDA receptor activity, Ca^2+^ influx and Erk1/2 phosphorylation. *Via* this route L-lactate could enhance expression of plasticity-related genes Arc, c-Fos, Zif268, and BDNF. These effects required high concentrations of L-lactate (10 to 20 mmol/L). Also note that there is evidence that extracellular acidification as may be expected when lactate builds up in the tissue, inhibits NMDA receptors (see text for details). Lac, L-lactate; Pyr, pyruvate.

In a (CHO)-K1 cell line expressing GPR81, sodium L-lactate concentration dependently increased binding of ^35^S-GTPγS (EC_50_≈1.3 mmol/L) and inhibited forskolin-stimulated cAMP production.^55^ Furthermore, the activating effect of L-lactate on GPR81 was confirmed in a calcium mobilization assay in CHO cells stably co-expressing GPR81 with the G_qi5_ protein which promiscuously couples various GPCRs to the phospholipase C-IP_3_ pathway. Pretreatment of the cells with a G_i_ protein inactivator, pertussis toxin, completely abolished the effect of L-lactate-mediated activation of GPR81 in the ^35^S-GTPγS binding assay. Interestingly, in the same assay the stereoisomer D-lactate also showed the ability to activate GPR81 although with a higher EC_50_ (~3 mmol/L). These *in vitro* experiments clearly showed that L-lactate is able to stimulate G_i_-coupled GPR81, causing inhibition of cAMP-mediated intracellular signaling events (Figure 2A).

GPR81 is enriched in adipose tissue and was originally proposed as a potential target for treatment of dyslipidemia.^55, 57^ The initial studies indicated a negligible level of GPR81 in the brain as compared with adipocytes. However, a follow-up study by Lauritzen *et al*^58^ suggested that GPR81 (HCA1) is nevertheless expressed in the rat brain at about 100-fold lower levels than in adipose tissue. The most prominent labeling was found in Purkinje cells in cerebellum, in pyramidal cells in hippocampus, in neurons of the dentate hilus, and in neocortex. Furthermore, at the mRNA and protein levels, GPR81 was detected in hippocampus and cerebellum and at a lower level in the cortex. Immunoreactivity for GPR81 was localized mostly in neurons and to a lesser extent in astrocytes with twice higher density at the plasma membranes of vascular endothelial cells, the luminal and abluminal leaflets than on the membranes of perivascular astrocytic endfeet.^58^ In human tissue, GPR81 mRNA expression was detected in pituitary gland but neither in frontal, temporal, and occipital lobes of the cortex, nor in forebrain, caudate nucleus, nucleus accumbens, and hippocampus.^54^ In mouse primary cortical neuronal cultures, its presence at the protein level was confirmed by immunohistochemistry and western blot.^59^

Consistent with an effect mediated *via* a G_i_-coupled pathway (Figure 2A), forskolin-induced cAMP production in acute hippocampal slices could be concentration dependently inhibited by L-lactate but this required concentrations of 10 mmol/L and more.^58^ Even 30 mmol/L of L-lactate resulted in inhibition by less than 30%. Similar effects were elicited by a more recently identified GPR81 agonist, 3,5-dihydroxybenzolic acid.^56^ In line with these activity levels, Gilbert *et al*^60^ reported that high concentrations of L-lactate (application of 250 mmol/L solution *via* a local microdialysis probe) resulted in reversible suppression of the firing activity of hippocampal pyramidal cells *in vivo* although the authors explained their observations by a metabolic, rather than receptor-mediated actions.

Using calcium imaging in primary cortical cultures, Bozzo *et al*^59^ found that L-lactate inhibited the frequency of calcium transients by about 50% in both principal and GABAergic interneurons in a concentration-dependent manner (IC_50_~5 mmol/L) in the presence of 5 mmol/L glucose. Such an effect of L-lactate could not mimicked by the application of pyruvate or by high (10 mmol/L) or low (0.5 mmol/L) concentrations of glucose. Furthermore, pertussis toxin abolished the inhibitory effect of 10 mmol/L L-lactate on calcium transient frequency. 3,5-Dihydroxybenzolic acid as well as a nonspecific agonist of HCA1 and HCA2 receptors, 3-HBA, also inhibited calcium transients at concentrations of ~1 mmol/L.^59^

Taken together, HCA1 (GPR81) is obviously present in some parts of the CNS and its distribution is nonuniform. Given that all studies published to date consistently required applications of very high concentrations of L-lactate (5 mmol/L and more) for activation of HCA1, it may likely have a role under conditions of ischemia or extreme neuronal activity (for example, seizures).

### A Putative Excitatory G Protein-Coupled Receptor for L-lactate in the Locus Coeruleus

Recently, we have shown an excitatory effect of astrocyte-derived L-lactate on noradrenergic (NAergic) neurons in locus coeruleus (LC) (Figure 2B). Organotypic cultured slices were cotransfected with two viral vectors, one targeting astrocytes and carrying a channelrhodopsin-2 mutant, ChR2(H134R), the other targeting LC NAergic neurons for expression of DsRED fluorescent protein.^61^ This approach allowed us to selectively activate astrocytes, and carry out whole cell recordings from visualized LC neurons. After about 60 seconds of optogenetic stimulation of astrocytes with blue light we registered depolarization and increased action potential activity in NAergic neurons. This delayed depolarization could be prevented by treatments that should have prevented formation of L-lactate, for example by inhibiting LDH (by oxamate) or glycogen metabolism (by 1,4-dideoxy-1,4-imino-D-arabinitol). We also found that optogenetic stimulation led to acidification of cultured astrocytes, which could be prevented by blocking glycogen mobilization. We reasoned that the delayed depolarizations of LC neurons mentioned above could be a result of L-lactate release from astrocytes. In agreement with this hypothesis, a concentration-dependent excitatory effect was observed with exogenously applied L-lactate (0.2 to 6 mmol/L). Coapplication of D-lactate abolished also depolarization of NAergic neurons evoked by activation of astrocytes or L-lactate application. Interestingly, blockers of glutamate and P2Y receptors did not prevent the stimulatory effect, arguing against indirect effects of L-lactate. Several lines of evidence argue against the notion that the effect of L-lactate on LC neurons was metabolic (e.g., was a consequence of its use as an energy source). First, all experiments were performed with an excess of glucose (glucose concentration in bath 5.5 mmol/L; internal pipette solution contained 5 mmol/L glucose and 2 mmol/L ATP). Second, pyruvate did not mimic the L-lactate action and did not depolarize LC neurons. Third, when added to the patch pipette solution, L-lactate did not evoke membrane potential changes but when applied into the bath it still excited LC neurons. Finally, an MCT blocker 4-CIN was also ineffective. Interestingly, blockade of adenylate cyclase, protein kinase A, but not protein kinase C abolished the effect of L-lactate, consistent with c-AMP mediated signaling in LC neurons (Figure 2B). Furthermore, using fast scan voltammetry in organotypic and acute brain slices, we showed that LC neurons release NA in response to L-lactate application and to optogenetic activation of astrocytes and that these responses were also mediated by adenylate cyclase and protein kinase A (although in this case we cannot pinpoint the cell type affected by the drugs). *In vivo*, injection of L-lactate into the LC induced an increase in the power of the high frequency bands of the electroencephalography and an increase in arterial blood pressure, in line with increased NAergic activity in the cortex.

At present, we do not know the identity of the molecular mechanism behind these effects of L-lactate on NAergic neurons but all the data are consistent with the existence of a yet unknown excitatory GPCR for L-lactate (Figure 2B), possibly one of the orphan GPCRs or dimer of a known GPCR with HCA1. Whatever the molecular nature of this signaling cascade, it may create an interesting positive feedback loop between NAergic axons and astrocytes and in theory locally couple NAergic input to the activity and metabolic status of local neurons and astrocytes *via* L-lactate.

### K_ATP_ Channel-Mediated Effects of L-lactate in the Brain

As mentioned above, while the transfer of L-lactate from astrocytes to neurons is definitely possible, not all studies support the idea that such a transfer would represent a major benefit to neurons while glucose is available. Nevertheless, if astrocyte-derived L-lactate was indeed used to boost the concentration of ATP in neurons, this could create a situation similar to that in pancreatic β-cells, where ATP/ADP ratio is monitored by K_ATP_ channels. These channels close when cytoplasmic ATP levels rise as may be expected when the supply of energy substrates increases, leading to depolarization of the membrane.^62, 63^ K_ATP_ channels are present in the CNS^64, 65^ and some studies have implicated them in the effects of L-lactate on certain populations of neurons. Hypothalamic neurons and orexin neurons in particular serve as important central regulators of food intake.^66, 67^ These cells are known for their ability to sense changes in glucose.^68, 69^ They are unusual in that they seem to lack glucokinase^70^ making them more dependent on alternative fuels. Interestingly, some of the neurons in hypothalamus were shown to be sensitive to L-lactate and to respond to 5 mmol/L L-lactate with an elevation of intracellular ATP as well as excitation.^71, 72^ Parson *et al*^68^ showed that orexin neurons in the perifornical area lost their spontaneous firing activity within 20 minutes when glucose deprived (0 mmol/L glucose in extracellular media and internal solution), which could be restored by the addition of glucose or L-lactate in a concentration-dependent manner. In glucose-free conditions, inhibition of astrocyte metabolism by the glial toxin fluoroacetate prevented restoration of firing activity by glucose (1 to 2.5 mmol/L). Fluoroacetate did not affect the ability of L-lactate (2 to 5 mmol/L) to completely restore firing rate of orexin neurons. This effect points to the involvement of astrocyte-derived L-lactate in the rescuing effect of glucose. Using perforated patch-clamp recording these authors also showed that hyperpolarization of orexin neurons induced by withdrawal of glucose could be abolished by the K_ATP_ channel blocker, glibenclamide. In the presence of 2.5 mmol/L glucose in artificial cerebrospinal fluid as well as in internal solution spontaneous firing activity of orexin neurons could be reversibly abolished by 4-CIN, an inhibitor of MCTs. However, such a mechanism of glucose sensing seem to be not universal for orexin neurons as the study by Venner *et al*^69^ showed that orexin neurons respond with hyperpolarization to L-lactate infused into the cytosol (IC_50_~17.4 mmol/L) when extracellular glucose levels are increased up to 5 mmol/L. Furthermore, the glial toxin fluoroacetate in that study hyperpolarized orexin neurons in the presence of 5 mmol/L glucose in the bath. However, whether L-lactate sensing is ATP dependent in such conditions needs further investigation.

Overall, study by Parson *et al*^68^ put forward a case for a K_ATP_ channel-mediated effect of L-lactate to stimulate orexin neurons, which could be a unique feature of these cells (Figure 2C). This mechanism of L-lactate action is cell and region specific, requires L-lactate transport into the cytoplasm, and might be essential for brain monitoring of glucose levels.

### Modulation of NMDA Receptors by L-lactate

Recently, Yang *et al*^73^ reported that, *in vivo* and *in vitro*, L-lactate drives expression of the plasticity-related genes Arc, c-Fos, and Zif268 in neurons in a time- and concentration-dependent manner. *In vitro* expression was studied at mRNA level in primary cultures of mouse embryonic (E17) cortical neurons in media containing 25 mmol/L glucose. The threshold concentration of L-lactate to alter Arc expression was 2.5 mmol/L while 20 mmol/L L-lactate caused four to eightfold increases in Zif268, Arc, and c-Fos expression with the maximum increase detected 60 minutes after L-lactate application. The effect of L-lactate could be blocked by the MCT inhibitor UK5099 but was mimicked neither by application of glucose (20 mmol/L, a theoretically 'equicaloric' level to 10 mmol/L L-lactate, but note that both metabolites were in great excess of their physiologic concentrations), nor by pyruvate (20 mmol/L), nor the enantiomer D-lactate (10 mmol/L). Pretreatment with the NMDA receptor antagonist MK801 prevented the increase of Arc and Zif268 mRNA and protein by 20 mmol/L L-lactate. Furthermore, application of 20 mmol/L L-lactate but not pyruvate to neuronal cultures significantly increased the concentration of phosphorylated extracellular signal-regulated kinase (Erk1/2), which had been previously implicated in NMDA receptor signaling related plasticity. Blocking NMDA receptors or Erk1/2 phosphorylation abolished the effect of 20 mmol/L L-lactate on Arc and Zif268 levels. Whole cell recordings from primary cortical cultures revealed that L-lactate (10 and 20 mmol/L) enhanced inward currents evoked by coapplication of glutamate and glycine, an effect that could be abolished by MK801 (Figure 2D). Moreover, L-lactate (10 mmol/L) but not pyruvate potentiated Ca^2+^ increases in neurons, which were induced by stimulation of NMDA receptors using coapplication of glutamate and glycine. Studies published two decades ago reported that NR1 subunit of the NMDA receptor has cysteine residues, which make it potentially sensitive to reducing agents.^74, 75^ Therefore, Yang *et al*^73^ hypothesized that the effect of L-lactate could be mediated by a change in the redox state of the cells because transformation of the imported L-lactate into pyruvate leads to an increase in the NADH/NAD ratio. In fact, 20 mmol/L L-lactate but not pyruvate increased the intracellular NADH/NAD^+^ ratio by 2.5-fold. NADH (4 mmol/L) when added extracellularly increased Arc, Zif268, as well as Erk1/2 phosphorylation in a MK801-sensitive manner. In addition, application of NADH into the bath increased intracellular Ca^2+^ levels, which could be prevented by MK801. Finally, intracortical injections of L-lactate (10 mmol/L) but not D-lactate (10 mmol/L), nor pyruvate (10 mmol/L) into the sensory motor cortex of anesthetized mice resulted in increased expression of Arc, c-Fos, and Zif268 *in vivo*.

The study implies that L-lactate may affect activity of cortical neurons as a result of the shift in the NADH/NAD^+^ ratio, which follows L-lactate conversion into pyruvate (Figure 1D). Whether the proposed increases in NMDA receptor activity are the actual cause of Erk1/2 phosphorylation or are secondary to Ca^2+^ influx was not established. Interestingly, pyruvate that should have an opposite effect on the redox state was completely inactive in these experiments. Furthermore, export of lactate *via* MCTs results in increase in H+ concentration and acidification. Two early studies found that a decrease in extracellular pH inhibits NMDA-activated currents.^76, 77^ This seems to conflict with the idea of lactate being a positive modulator of NMDA receptor-mediated signaling.

It is also important to note that the observed effects were only significant from L-lactate concentrations of 2.5 mmol/L and above and most of the experiments were performed using 20 mmol/L L-lactate. As discussed above, it is highly unlikely that such concentrations can be reached in a healthy brain and the physiologic significance of this mechanism remains to be demonstrated. It is also not clear at what level these effects coexist with the inhibitory HCA1-mediated action of L-lactate demonstrated by the studies cited above.^55, 58, 59, 78, 79^ To note, one earlier study reported that high concentrations of L-lactate (250 to 500 mmol/L) applied *via* microdialysis inhibited rather than excited hippocampal neurons.^60^ One could speculate that an inhibitory action of L-lactate, for example *via* HCA1 (GPR81), might override the NMDA receptor-mediated effect, depending on the physiologic context.

## Conclusions

In this review, we have summarized some of the current ideas about a signaling role of L-lactate in the brain. The proposed mechanisms either pivot around the intracellular fate of L-lactate (contribution to ATP production or change in NADH/NAD^+^ ratio induced by L-lactate to pyruvate conversion) or the existence of a designated receptor for L-lactate, a mechanism that does not require L-lactate entry into the target neuron. In future experiments, it will be important to get a more precise understanding of the fluctuations of extracellular L-lactate concentration in brain microdomains to put the mechanisms described above into their physiologic context. At present it seems that some of them require unrealistically high concentrations of L-lactate but this view may change if we discover highly localized hot spots of L-lactate synthesis and release. Here, experiments with genetically encoded L-lactate sensors should be particularly informative.^80^ The idea of specific GPCR-mediated L-lactate signaling is attractive because there are numerous precedents of cognate receptors for other metabolites (e.g., HCA2 and HCA3) or for protons (GPR4 and others) present in the brain. It can also be hypothesized that since release of L-lactate *via* MCT is inevitably accompanied by protons and by local acidification, these proton receptors would be (co-)activated, even if the effect may not then be selective for L-lactate. There is still much to learn about the physiology of the long-standing metabolite L-lactate in the brain.
